# Supplementary material for: Genome-Wide Analysis of the Odorant Receptor Gene Family in Solenopsis invicta, Ooceraea biroi, and Monomorium pharaonis (Hymenoptera: Formicidae)
Source: Int J Mol Sci. 2023 Apr 1;24(7):6624. doi: 10.3390/ijms24076624 (PMC10095046; doi:10.3390/ijms24076624)
Supplement: Supplementary file 1 [file ijms-24-06624-s001.zip › ijms-2266728-supplementary.pdf]

# Supplementary Material

Genome-wide Analysis of the Odorant Receptor Gene Family in *Solenopsis invicta*, *Ooceraea biroi*, *and Monomorium pharaonis* (Hymenoptera: Formicidae)

Bo Zhang[1,2,3#], Rong-Rong Yang[4,#], Xingchuan Jiang[3], Xiaoxia Xu[4], Bing Wang[1*], Gui-Rong Wang[1, 2*]

[1] State Key Laboratory for Biology of Plant Diseases and Insect Pests, Institute of Plant Protection, Chinese Academy of Agricultural Sciences, Beijing 100193, China

[2] Shenzhen Branch, Guangdong Laboratory for Lingnan Modern Agriculture, Genome Analysis Laboratory of the Ministry of Agriculture, Agricultural Genomics Institute at Shenzhen, Chinese Academy of Agricultural Sciences, Shenzhen 518120, China

[3] College of Plant Protection, Anhui Agricultural University, Hefei, Anhui 230036, China

[4] Laboratory of Bio-Pesticide Creation and Application of Guangdong Province, College of Plant Protection, South China Agricultural University, Guangzhou 510642, China

# These authors contributed equally to this work.

*Correspondence: wangguirong@caas.cn (Guirong Wang); wangbing02@caas.cn (Bing Wang)

# 1 Supplementary Figures and Tables

## 1.1 Supplementary Tables

**Table S1** Genomic information of three Formicidae species.

| Feature | *Ooceraea biroi* | *Monomorium pharaonis* | *Solenopsis invicta* |
|---|---|---|---|
| Genome Size (Mb) | 216 | 314 | 365 |
| Assembly level | chromosome | chromosome | chromosome |
| Numbers of contigs | 535 | 1172 | 317 |
| Numbers of scaffolds | 139 | 725 | 219 |
| Scaffold N50 (Mb) | 16.89 | 29.66 | 26.22 |
| BUSCO genes (%) | C:98.5 [S:96.2, D:2.3], F:0.6, M:0.9 | C:98.9 [S:95.2, D:3.7], F:0.3, M:0.8 | C:99.1 [S:97.4, D:1.7], F:0.3, M:0.6 |
| GC Content (%) | 41.31 | 36.37 | 36.38 |

Note: S: Complete and single-copy BUSCOs; D: Complete and duplicated BUSCOs; F: Fragmented BUSCOs; M: Missing BUSCOs.

**Table S2** Odorant receptor genes of *S. invicta* experienced tandem replication.

| Tandem Duplication | | | |
|---|---|---|---|
| SinvOR4 | SinvOR5 | SinvOR158 | SinvOR159 |
| SinvOR5 | SinvOR6 | SinvOR164 | SinvOR165 |
| SinvOR6 | SinvOR7 | SinvOR165 | SinvOR166 |
| SinvOR7 | SinvOR8 | SinvOR170 | SinvOR171 |
| SinvOR14 | SinvOR15 | SinvOR180 | SinvOR181 |
| SinvOR18 | SinvOR19 | SinvOR185 | SinvOR186 |
| SinvOR20 | SinvOR21 | SinvOR186 | SinvOR187 |
| SinvOR21 | SinvOR22 | SinvOR205 | SinvOR206 |
| SinvOR24 | SinvOR25 | SinvOR209 | SinvOR210 |
| SinvOR34 | SinvOR35 | SinvOR226 | SinvOR227 |
| SinvOR35 | SinvOR36 | SinvOR227 | SinvOR228 |
| SinvOR36 | SinvOR37 | SinvOR230 | SinvOR321 |
| SinvOR40 | SinvOR41 | SinvOR234 | SinvOR235 |
| SinvOR43 | SinvOR44 | SinvOR238 | SinvOR239 |
| SinvOR49 | SinvOR50 | SinvOR240 | SinvOR241 |
| SinvOR55 | SinvOR56 | SinvOR242 | SinvOR243 |
| SinvOR73 | SinvOR74 | SinvOR248 | SinvOR249 |
| SinvOR81 | SinvOR82 | SinvOR253 | SinvOR254 |
| SinvOR89 | SinvOR90 | SinvOR262 | SinvOR263 |
| SinvOR93 | SinvOR94 | SinvOR271 | SinvOR272 |
| SinvOR99 | SinvOR100 | SinvOR276 | SinvOR277 |
| SinvOR100 | SinvOR101 | SinvOR277 | SinvOR278 |
| SinvOR109 | SinvOR110 | SinvOR279 | SinvOR280 |
| SinvOR111 | SinvOR112 | SinvOR288 | SinvOR289 |
| SinvOR112 | SinvOR113 | SinvOR300 | SinvOR301 |
| SinvOR116 | SinvOR117 | SinvOR302 | SinvOR303 |
| SinvOR120 | SinvOR121 | SinvOR311 | SinvOR312 |
| SinvOR122 | SinvOR123 | SinvOR313 | SinvOR314 |
| SinvOR124 | SinvOR125 | SinvOR314 | SinvOR315 |
| SinvOR125 | SinvOR126 | SinvOR316 | SinvOR317 |
| SinvOR138 | SinvOR139 | SinvOR331 | SinvOR332 |
| SinvOR139 | SinvOR140 | SinvOR334 | SinvOR335 |
| SinvOR142 | SinvOR143 | SinvOR338 | SinvOR339 |
| SinvOR143 | SinvOR144 | SinvOR342 | SinvOR343 |
| SinvOR150 | SinvOR151 | SinvOR344 | SinvOR345 |
| SinvOR157 | SinvOR158 | | |

Note: MCscanX defined tandem duplicates as paralogs that are adjacent to each other on chromosomes, which are suggested to arise from illegitimate chromosomal recombination. In any BLASTP hit, the two genes are re-labeled as 'tandem duplicates' if they have a difference of gene rank = 1(Wang et al., 2012).

**Table S3** DEG analysis of *ORs* in antennae of *S. invicta* workers between monogyne and polygyne

| ID | polygyne | monogyne | baseMean | log2FC | pvalue | FDR |
|---|---|---|---|---|---|---|
| SinvOrco | 1420.34 | 1846.93 | 1633.64 | -0.38 | 3.08E-01 | 7.78E-02 |
| SinvOR1 | 2.55 | 3.14 | 2.84 | -0.31 | 7.69E-01 | 5.72E-01 |
| SinvOR2 | 2.58 | 0.37 | 1.47 | 2.96 | 1.41E-01 | 1.30E-02 |
| SinvOR3 | 2.70 | 4.32 | 3.51 | -0.68 | 6.51E-01 | 3.86E-01 |
| SinvOR4 | 12.00 | 12.34 | 12.17 | -0.04 | 9.27E-01 | 8.47E-01 |
| SinvOR5 | 19.35 | 11.15 | 15.25 | 0.77 | 2.78E-01 | 6.24E-02 |
| SinvOR6 | 6.88 | 6.84 | 6.86 | -0.09 | 8.95E-01 | 7.96E-01 |
| SinvOR7 | 7.27 | 8.96 | 8.12 | -0.34 | 6.73E-01 | 4.29E-01 |
| SinvOR8 | 11.66 | 8.23 | 9.94 | 0.42 | 4.36E-01 | 1.58E-01 |
| SinvOR9 | 10.18 | 0.00 | 5.09 | 5.65 | 1.00E+00 | 9.94E-01 |
| SinvOR10 | 45.87 | 38.26 | 42.07 | 0.28 | 1.92E-01 | 2.65E-02 |
| SinvOR11 | 21.70 | 19.17 | 20.44 | 0.20 | 5.52E-01 | 2.65E-01 |
| SinvOR12 | 26.46 | 25.55 | 26.00 | 0.07 | 8.87E-01 | 7.75E-01 |
| SinvOR13 | 15.80 | 20.36 | 18.08 | -0.35 | 3.01E-01 | 7.51E-02 |
| SinvOR14 | 36.12 | 35.01 | 35.56 | 0.01 | 9.67E-01 | 9.32E-01 |
| SinvOR15 | 19.58 | 24.17 | 21.88 | -0.34 | 2.90E-01 | 6.88E-02 |
| SinvOR16 | 39.36 | 24.53 | 31.94 | 0.73 | 1.53E-01 | 1.55E-02 |
| SinvOR17 | 13.95 | 9.42 | 11.69 | 0.48 | 3.27E-01 | 8.97E-02 |
| SinvOR18 | 46.67 | 34.55 | 40.61 | 0.38 | 1.81E-01 | 2.34E-02 |
| SinvOR19 | 36.15 | 17.89 | 27.02 | 1.03 | 5.12E-01 | 2.30E-01 |
| SinvOR20 | 8.85 | 5.64 | 7.24 | 0.63 | 2.89E-01 | 6.67E-02 |
| SinvOR21 | 8.18 | 0.39 | 4.29 | 3.99 | 1.87E-02 | 3.45E-04 |
| SinvOR22 | 10.60 | 9.24 | 9.92 | 0.18 | 7.65E-01 | 5.63E-01 |
| SinvOR23 | 14.16 | 11.17 | 12.66 | 0.26 | 6.21E-01 | 3.44E-01 |
| SinvOR24 | 19.80 | 15.32 | 17.56 | 0.40 | 3.71E-01 | 1.20E-01 |
| SinvOR25 | 8.00 | 10.22 | 9.11 | -0.35 | 7.71E-01 | 5.79E-01 |
| SinvOR27 | 18.94 | 13.95 | 16.44 | 0.45 | 3.91E-01 | 1.33E-01 |
| SinvOR28 | 15.40 | 19.00 | 17.20 | -0.27 | 4.79E-01 | 1.97E-01 |
| SinvOR29 | 55.27 | 67.68 | 61.47 | -0.32 | 1.75E-01 | 2.05E-02 |
| SinvOR30 | 78.91 | 63.65 | 71.28 | 0.29 | 3.34E-01 | 9.56E-02 |
| SinvOR32 | 16.22 | 13.43 | 14.83 | 0.23 | 6.65E-01 | 4.20E-01 |
| SinvOR33 | 59.84 | 71.36 | 65.60 | -0.27 | 2.52E-01 | 4.89E-02 |
| SinvOR34 | 40.62 | 46.09 | 43.35 | -0.19 | 5.74E-01 | 2.89E-01 |
| SinvOR35 | 2.74 | 19.10 | 10.92 | -2.83 | 1.81E-02 | 2.79E-04 |
| SinvOR36 | 12.05 | 10.98 | 11.52 | 0.16 | 7.47E-01 | 5.38E-01 |
| SinvOR37 | 70.14 | 66.06 | 68.10 | 0.08 | 7.11E-01 | 4.83E-01 |
| SinvOR38 | 27.49 | 34.55 | 31.02 | -0.34 | 2.11E-01 | 3.19E-02 |
| SinvOR39 | 13.48 | 3.12 | 8.30 | 2.20 | 1.46E-01 | 1.39E-02 |
| SinvOR40 | 51.76 | 47.70 | 49.73 | 0.10 | 6.83E-01 | 4.47E-01 |

| | | | | | | |
|---|---|---|---|---|---|---|
| SinvOR41 | 6.44 | 6.03 | 6.24 | 0.06 | 9.25E-01 | 8.43E-01 |
| SinvOR43 | 11.22 | 11.85 | 11.53 | -0.03 | 9.60E-01 | 9.10E-01 |
| SinvOR44 | 17.09 | 17.72 | 17.40 | -0.06 | 9.67E-01 | 9.28E-01 |
| SinvOR45 | 69.73 | 51.34 | 60.53 | 0.44 | 5.85E-01 | 3.02E-01 |
| SinvOR46 | 81.82 | 79.01 | 80.42 | 0.07 | 7.66E-01 | 5.66E-01 |
| SinvOR47 | 31.51 | 32.49 | 32.00 | -0.01 | 9.83E-01 | 9.65E-01 |
| SinvOR48 | 31.43 | 43.55 | 37.49 | -0.46 | 2.46E-01 | 4.46E-02 |
| SinvOR49 | 59.30 | 58.65 | 58.97 | 0.01 | 9.76E-01 | 9.49E-01 |
| SinvOR50 | 125.89 | 106.33 | 116.11 | 0.23 | 3.64E-01 | 1.14E-01 |
| SinvOR51 | 74.70 | 74.77 | 74.73 | -0.03 | 8.93E-01 | 7.91E-01 |
| SinvOR52 | 20.29 | 8.48 | 14.39 | 1.25 | 6.44E-01 | 3.74E-01 |
| SinvOR53 | 1.72 | 1.91 | 1.82 | -0.12 | 9.33E-01 | 8.64E-01 |
| SinvOR54 | 3.49 | 3.13 | 3.31 | 0.12 | 8.92E-01 | 7.88E-01 |
| SinvOR55 | 12.76 | 23.70 | 18.23 | -0.89 | 1.77E-01 | 2.18E-02 |
| SinvOR56 | 96.74 | 80.27 | 88.51 | 0.26 | 1.09E-01 | 7.01E-03 |
| SinvOR57 | 57.65 | 59.96 | 58.81 | -0.04 | 8.67E-01 | 7.31E-01 |
| SinvOR58 | 154.58 | 225.21 | 189.90 | -0.54 | 9.88E-02 | 5.77E-03 |
| SinvOR60 | 118.71 | 125.25 | 121.98 | -0.06 | 7.21E-01 | 4.99E-01 |
| SinvOR61 | 2.44 | 3.11 | 2.78 | -0.34 | 7.17E-01 | 4.92E-01 |
| SinvOR62 | 8.80 | 10.83 | 9.81 | -0.27 | 7.39E-01 | 5.25E-01 |
| SinvOR64 | 18.64 | 15.58 | 17.11 | 0.25 | 8.61E-01 | 7.21E-01 |
| SinvOR65 | 14.02 | 11.83 | 12.93 | 0.23 | 6.39E-01 | 3.68E-01 |
| SinvOR66 | 4.64 | 3.51 | 4.07 | 0.36 | 6.55E-01 | 3.97E-01 |
| SinvOR67 | 3.66 | 7.10 | 5.38 | -0.91 | 4.77E-01 | 1.94E-01 |
| SinvOR68 | 8.49 | 2.90 | 5.70 | 1.52 | 1.00E+00 | 9.97E-01 |
| SinvOR69 | 0.23 | 1.36 | 0.80 | -2.35 | 4.96E-01 | 2.15E-01 |
| SinvOR70 | 6.27 | 10.48 | 8.38 | -0.75 | 4.88E-01 | 2.06E-01 |
| SinvOR71 | 12.96 | 14.82 | 13.89 | -0.19 | 9.31E-01 | 8.59E-01 |
| SinvOR72 | 29.38 | 20.74 | 25.06 | 0.48 | 2.34E-01 | 3.97E-02 |
| SinvOR73 | 11.27 | 15.80 | 13.53 | -0.45 | 5.92E-01 | 3.11E-01 |
| SinvOR74 | 23.11 | 21.92 | 22.51 | 0.07 | 8.47E-01 | 6.88E-01 |
| SinvOR75 | 11.78 | 7.42 | 9.60 | 0.63 | 6.53E-01 | 3.94E-01 |
| SinvOR76 | 21.56 | 28.12 | 24.84 | -0.33 | 2.39E-01 | 4.20E-02 |
| SinvOR77 | 32.37 | 30.24 | 31.30 | 0.05 | 8.69E-01 | 7.35E-01 |
| SinvOR78 | 93.50 | 67.69 | 80.59 | 0.47 | 2.38E-01 | 4.09E-02 |
| SinvOR79 | 155.60 | 168.97 | 162.29 | -0.11 | 6.02E-01 | 3.28E-01 |
| SinvOR80 | 14.55 | 16.05 | 15.30 | -0.15 | 6.91E-01 | 4.59E-01 |
| SinvOR81 | 17.71 | 13.26 | 15.49 | 0.41 | 2.70E-01 | 5.65E-02 |
| SinvOR82 | 14.37 | 10.91 | 12.64 | 0.41 | 3.56E-01 | 1.08E-01 |
| SinvOR83 | 30.12 | 22.87 | 26.50 | 0.39 | 1.39E-01 | 1.24E-02 |
| SinvOR84 | 70.05 | 61.24 | 65.64 | 0.17 | 3.81E-01 | 1.27E-01 |
| SinvOR86 | 5.71 | 9.50 | 7.61 | -0.76 | 2.60E-01 | 5.12E-02 |

| | | | | | | |
|---|---|---|---|---|---|---|
| SinvOR87 | 15.13 | 15.65 | 15.39 | -0.11 | 8.35E-01 | 6.63E-01 |
| SinvOR88 | 30.27 | 31.98 | 31.12 | -0.09 | 7.63E-01 | 5.59E-01 |
| SinvOR89 | 70.33 | 60.00 | 65.17 | 0.20 | 3.34E-01 | 9.34E-02 |
| SinvOR90 | 12.79 | 6.02 | 9.40 | 1.02 | 1.06E-01 | 6.54E-03 |
| SinvOR91 | 32.64 | 35.53 | 34.08 | -0.11 | 7.63E-01 | 5.56E-01 |
| SinvOR92 | 13.12 | 10.51 | 11.81 | 0.32 | 5.03E-01 | 2.20E-01 |
| SinvOR93 | 8.22 | 14.06 | 11.14 | -0.79 | 7.41E-02 | 3.65E-03 |
| SinvOR94 | 5.61 | 8.98 | 7.30 | -0.68 | 2.46E-01 | 4.55E-02 |
| SinvOR95 | 4.96 | 11.61 | 8.29 | -1.22 | 1.84E-01 | 2.50E-02 |
| SinvOR96 | 3.81 | 10.79 | 7.30 | -1.50 | 3.33E-01 | 9.23E-02 |
| SinvOR97 | 11.19 | 13.70 | 12.44 | -0.33 | 5.05E-01 | 2.22E-01 |
| SinvOR98 | 8.66 | 10.74 | 9.70 | -0.28 | 6.49E-01 | 3.82E-01 |
| SinvOR99 | 28.37 | 42.68 | 35.52 | -0.55 | 3.79E-02 | 1.28E-03 |
| SinvOR100 | 30.40 | 38.42 | 34.41 | -0.28 | 2.78E-01 | 6.33E-02 |
| SinvOR101 | 67.26 | 84.76 | 76.01 | -0.31 | 1.32E-01 | 1.05E-02 |
| SinvOR102 | 93.34 | 88.96 | 91.15 | 0.08 | 7.23E-01 | 5.05E-01 |
| SinvOR104 | 58.64 | 66.89 | 62.77 | -0.16 | 5.35E-01 | 2.49E-01 |
| SinvOR105 | 6.58 | 10.73 | 8.66 | -0.71 | 6.07E-01 | 3.32E-01 |
| SinvOR106 | 15.94 | 15.54 | 15.74 | -0.01 | 9.91E-01 | 9.82E-01 |
| SinvOR107 | 33.56 | 33.12 | 33.34 | -0.01 | 9.62E-01 | 9.18E-01 |
| SinvOR108 | 36.25 | 32.13 | 34.19 | 0.20 | 4.71E-01 | 1.88E-01 |
| SinvOR109 | 2.12 | 2.91 | 2.51 | -0.48 | 8.84E-01 | 7.61E-01 |
| SinvOR110 | 19.28 | 15.44 | 17.36 | 0.24 | 5.52E-01 | 2.67E-01 |
| SinvOR111 | 18.49 | 17.70 | 18.10 | 0.08 | 8.11E-01 | 6.34E-01 |
| SinvOR113 | 11.95 | 9.14 | 10.54 | 0.40 | 8.85E-01 | 7.65E-01 |
| SinvOR114 | 28.48 | 26.54 | 27.51 | 0.06 | 8.45E-01 | 6.79E-01 |
| SinvOR115 | 8.87 | 55.32 | 32.10 | -2.63 | 1.99E-01 | 2.87E-02 |
| SinvOR116 | 39.34 | 37.98 | 38.66 | 0.09 | 7.96E-01 | 6.10E-01 |
| SinvOR117 | 76.01 | 90.66 | 83.34 | -0.22 | 3.14E-01 | 8.21E-02 |
| SinvOR118 | 0.69 | 2.55 | 1.62 | -1.92 | 4.53E-01 | 1.70E-01 |
| SinvOR119 | 21.02 | 18.00 | 19.51 | 0.17 | 6.40E-01 | 3.70E-01 |
| SinvOR120 | 95.78 | 113.91 | 104.84 | -0.24 | 3.90E-01 | 1.32E-01 |
| SinvOR121 | 33.39 | 27.19 | 30.29 | 0.34 | 3.23E-01 | 8.65E-02 |
| SinvOR122 | 15.98 | 12.67 | 14.32 | 0.29 | 4.79E-01 | 1.96E-01 |
| SinvOR123 | 20.84 | 20.83 | 20.84 | -0.04 | 9.05E-01 | 8.10E-01 |
| SinvOR124 | 13.20 | 5.77 | 9.49 | 1.20 | 2.90E-01 | 6.78E-02 |
| SinvOR126 | 14.50 | 14.42 | 14.46 | 0.03 | 9.43E-01 | 8.76E-01 |
| SinvOR127 | 20.45 | 22.91 | 21.68 | -0.15 | 6.80E-01 | 4.44E-01 |
| SinvOR128 | 3.60 | 2.77 | 3.19 | 0.35 | 8.92E-01 | 7.85E-01 |
| SinvOR129 | 34.85 | 33.65 | 34.25 | 0.01 | 9.77E-01 | 9.53E-01 |
| SinvOR130 | 0.00 | 2.92 | 1.46 | -4.16 | 1.76E-01 | 2.12E-02 |
| SinvOR131 | 0.50 | 1.35 | 0.93 | -1.52 | 4.91E-01 | 2.10E-01 |

| | | | | | | |
|---|---|---|---|---|---|---|
| SinvOR132 | 8.53 | 13.79 | 11.16 | -0.67 | 3.72E-01 | 1.22E-01 |
| SinvOR133 | 39.66 | 24.76 | 32.21 | 0.62 | 2.36E-02 | 5.82E-04 |
| SinvOR134 | 22.27 | 12.33 | 17.30 | 0.77 | 1.67E-01 | 1.74E-02 |
| SinvOR136 | 0.43 | 0.76 | 0.60 | -0.80 | 6.94E-01 | 4.63E-01 |
| SinvOR137 | 12.44 | 10.30 | 11.37 | 0.28 | 6.60E-01 | 4.10E-01 |
| SinvOR138 | 8.81 | 4.89 | 6.85 | 0.85 | 1.84E-01 | 2.44E-02 |
| SinvOR139 | 12.85 | 10.44 | 11.64 | 0.28 | 6.18E-01 | 3.40E-01 |
| SinvOR140 | 15.03 | 13.04 | 14.04 | 0.23 | 5.75E-01 | 2.92E-01 |
| SinvOR142 | 19.53 | 14.88 | 17.21 | 0.35 | 3.34E-01 | 9.45E-02 |
| SinvOR143 | 14.46 | 16.00 | 15.23 | -0.14 | 9.46E-01 | 8.85E-01 |
| SinvOR144 | 10.54 | 6.64 | 8.59 | 0.76 | 3.57E-01 | 1.10E-01 |
| SinvOR145 | 11.72 | 10.33 | 11.03 | 0.20 | 6.75E-01 | 4.32E-01 |
| SinvOR146 | 13.70 | 14.38 | 14.04 | -0.07 | 8.70E-01 | 7.38E-01 |
| SinvOR147 | 2.47 | 0.98 | 1.72 | 1.24 | 5.50E-01 | 2.59E-01 |
| SinvOR148 | 3.68 | 3.98 | 3.83 | -0.09 | 9.63E-01 | 9.21E-01 |
| SinvOR149 | 18.07 | 21.62 | 19.85 | -0.19 | 6.49E-01 | 3.79E-01 |
| SinvOR150 | 30.19 | 27.63 | 28.91 | 0.13 | 6.78E-01 | 4.40E-01 |
| SinvOR151 | 7.83 | 11.39 | 9.61 | -0.60 | 3.55E-01 | 1.07E-01 |
| SinvOR152 | 4.74 | 6.10 | 5.42 | -0.36 | 6.78E-01 | 4.38E-01 |
| SinvOR153 | 0.00 | 6.19 | 3.10 | -5.21 | 1.25E-01 | 9.65E-03 |
| SinvOR154 | 6.77 | 9.17 | 7.97 | -0.42 | 5.11E-01 | 2.26E-01 |
| SinvOR156 | 3.49 | 9.46 | 6.48 | -1.44 | 4.60E-01 | 1.75E-01 |
| SinvOR157 | 1.16 | 3.52 | 2.34 | -1.64 | 5.11E-01 | 2.28E-01 |
| SinvOR158 | 12.13 | 10.53 | 11.33 | 0.18 | 6.76E-01 | 4.35E-01 |
| SinvOR164 | 23.65 | 17.71 | 20.68 | 0.39 | 2.24E-01 | 3.59E-02 |
| SinvOR165 | 12.43 | 7.29 | 9.86 | 0.77 | 1.74E-01 | 1.98E-02 |
| SinvOR166 | 19.93 | 6.62 | 13.28 | 1.47 | 1.27E-03 | 3.91E-06 |
| SinvOR167 | 9.50 | 13.30 | 11.40 | -0.50 | 2.29E-01 | 3.73E-02 |
| SinvOR168 | 8.44 | 6.64 | 7.54 | 0.39 | 4.83E-01 | 2.01E-01 |
| SinvOR169 | 4.63 | 5.10 | 4.87 | -0.14 | 8.50E-01 | 6.93E-01 |
| SinvOR171 | 10.11 | 11.88 | 11.00 | -0.24 | 5.98E-01 | 3.20E-01 |
| SinvOR172 | 15.94 | 14.13 | 15.03 | 0.14 | 7.40E-01 | 5.28E-01 |
| SinvOR173 | 126.44 | 129.09 | 127.77 | -0.01 | 9.54E-01 | 8.98E-01 |
| SinvOR174 | 10.15 | 7.72 | 8.93 | 0.37 | 6.02E-01 | 3.26E-01 |
| SinvOR175 | 16.67 | 17.06 | 16.87 | -0.01 | 9.72E-01 | 9.39E-01 |
| SinvOR176 | 34.84 | 37.99 | 36.42 | -0.13 | 6.24E-01 | 3.53E-01 |
| SinvOR177 | 24.58 | 25.96 | 25.27 | -0.13 | 6.51E-01 | 3.84E-01 |
| SinvOR178 | 10.78 | 15.28 | 13.03 | -0.51 | 2.48E-01 | 4.65E-02 |
| SinvOR179 | 23.04 | 21.09 | 22.07 | 0.08 | 8.01E-01 | 6.18E-01 |
| SinvOR180 | 22.25 | 28.23 | 25.24 | -0.30 | 4.50E-01 | 1.66E-01 |
| SinvOR181 | 36.91 | 42.59 | 39.75 | -0.18 | 4.26E-01 | 1.52E-01 |
| SinvOR183 | 17.19 | 16.41 | 16.80 | 0.11 | 7.61E-01 | 5.53E-01 |

| | | | | | | |
|---|---|---|---|---|---|---|
| SinvOR184 | 23.07 | 25.60 | 24.33 | -0.10 | 7.29E-01 | 5.14E-01 |
| SinvOR185 | 17.44 | 14.15 | 15.80 | 0.29 | 8.54E-01 | 7.04E-01 |
| SinvOR186 | 36.78 | 45.32 | 41.05 | -0.26 | 4.66E-01 | 1.81E-01 |
| SinvOR187 | 67.10 | 106.32 | 86.71 | -0.65 | 1.92E-01 | 2.72E-02 |
| SinvOR188 | 17.30 | 20.18 | 18.74 | -0.22 | 5.93E-01 | 3.16E-01 |
| SinvOR189 | 14.18 | 21.58 | 17.88 | -0.58 | 1.17E-01 | 8.27E-03 |
| SinvOR190 | 13.07 | 17.86 | 15.47 | -0.46 | 6.55E-01 | 3.99E-01 |
| SinvOR191 | 18.93 | 28.55 | 23.74 | -0.55 | 1.13E-01 | 7.68E-03 |
| SinvOR193 | 18.18 | 3.97 | 11.07 | 2.22 | 3.82E-01 | 1.28E-01 |
| SinvOR194 | 20.04 | 26.12 | 23.08 | -0.34 | 2.33E-01 | 3.87E-02 |
| SinvOR195 | 1.47 | 7.64 | 4.55 | -2.46 | 3.42E-02 | 1.05E-03 |
| SinvOR196 | 0.00 | 14.53 | 7.26 | -6.44 | 5.73E-02 | 2.47E-03 |
| SinvOR199 | 17.65 | 22.31 | 19.98 | -0.29 | 4.53E-01 | 1.71E-01 |
| SinvOR200 | 17.34 | 14.51 | 15.92 | 0.22 | 5.90E-01 | 3.09E-01 |
| SinvOR201 | 19.83 | 20.95 | 20.39 | -0.03 | 9.29E-01 | 8.51E-01 |
| SinvOR202 | 22.16 | 9.01 | 15.59 | 1.22 | 1.19E-01 | 8.75E-03 |
| SinvOR203 | 33.60 | 27.66 | 30.63 | 0.25 | 6.60E-01 | 4.08E-01 |
| SinvOR204 | 20.41 | 21.14 | 20.77 | -0.04 | 9.01E-01 | 8.04E-01 |
| SinvOR205 | 23.79 | 24.31 | 24.05 | 0.01 | 9.87E-01 | 9.72E-01 |
| SinvOR206 | 14.53 | 15.63 | 15.08 | -0.09 | 9.07E-01 | 8.15E-01 |
| SinvOR207 | 41.95 | 48.60 | 45.27 | -0.21 | 4.66E-01 | 1.82E-01 |
| SinvOR208 | 5.66 | 9.44 | 7.55 | -0.69 | 2.94E-01 | 7.06E-02 |
| SinvOR209 | 0.74 | 1.97 | 1.36 | -1.45 | 6.67E-01 | 4.23E-01 |
| SinvOR210 | 3.06 | 2.14 | 2.60 | 0.54 | 5.93E-01 | 3.14E-01 |
| SinvOR211 | 4.13 | 2.53 | 3.33 | 0.58 | 5.82E-01 | 2.99E-01 |
| SinvOR212 | 9.48 | 5.09 | 7.28 | 0.77 | 2.73E-01 | 5.79E-02 |
| SinvOR213 | 2.57 | 2.14 | 2.35 | 0.24 | 9.30E-01 | 8.56E-01 |
| SinvOR214 | 1.17 | 2.58 | 1.87 | -1.16 | 6.64E-01 | 4.17E-01 |
| SinvOR215 | 2.43 | 1.97 | 2.20 | 0.27 | 7.96E-01 | 6.13E-01 |
| SinvOR216 | 5.94 | 5.13 | 5.53 | 0.20 | 9.48E-01 | 8.90E-01 |
| SinvOR217 | 22.07 | 21.08 | 21.58 | 0.05 | 8.70E-01 | 7.41E-01 |
| SinvOR218 | 13.90 | 11.32 | 12.61 | 0.27 | 5.54E-01 | 2.69E-01 |
| SinvOR219 | 20.85 | 23.46 | 22.15 | -0.12 | 7.41E-01 | 5.31E-01 |
| SinvOR220 | 30.64 | 35.52 | 33.08 | -0.18 | 6.84E-01 | 4.50E-01 |
| SinvOR221 | 43.70 | 36.46 | 40.08 | 0.29 | 2.69E-01 | 5.54E-02 |
| SinvOR222 | 31.09 | 30.21 | 30.65 | 0.06 | 8.90E-01 | 7.81E-01 |
| SinvOR223 | 6.71 | 0.00 | 3.35 | 5.04 | 1.37E-01 | 1.18E-02 |
| SinvOR224 | 7.05 | 6.48 | 6.76 | 0.18 | 8.04E-01 | 6.23E-01 |
| SinvOR227 | 8.40 | 3.94 | 6.17 | 0.98 | 3.54E-01 | 1.06E-01 |
| SinvOR228 | 10.94 | 11.94 | 11.44 | -0.15 | 7.36E-01 | 5.21E-01 |
| SinvOR229 | 46.14 | 37.35 | 41.74 | 0.27 | 2.43E-01 | 4.34E-02 |
| SinvOR230 | 29.49 | 32.36 | 30.92 | -0.14 | 5.88E-01 | 3.06E-01 |

| | | | | | | |
|---|---|---|---|---|---|---|
| SinvOR231 | 29.24 | 24.17 | 26.70 | 0.23 | 4.07E-01 | 1.44E-01 |
| SinvOR232 | 14.21 | 6.93 | 10.57 | 1.03 | 3.40E-01 | 1.00E-01 |
| SinvOR233 | 9.93 | 5.71 | 7.82 | 0.79 | 8.08E-01 | 6.29E-01 |
| SinvOR234 | 5.21 | 6.05 | 5.63 | -0.24 | 7.11E-01 | 4.81E-01 |
| SinvOR235 | 0.00 | 6.87 | 3.44 | -5.38 | 1.00E+00 | 1.00E+00 |
| SinvOR236 | 2.15 | 2.18 | 2.17 | 0.03 | 9.76E-01 | 9.46E-01 |
| SinvOR237 | 7.95 | 8.76 | 8.35 | -0.12 | 8.41E-01 | 6.73E-01 |
| SinvOR238 | 0.00 | 5.26 | 2.63 | -5.00 | 6.80E-02 | 3.14E-03 |
| SinvOR239 | 17.36 | 21.07 | 19.21 | -0.29 | 5.21E-01 | 2.37E-01 |
| SinvOR240 | 38.75 | 37.12 | 37.93 | 0.04 | 8.86E-01 | 7.71E-01 |
| SinvOR241 | 27.47 | 51.24 | 39.36 | -0.87 | 5.45E-03 | 3.35E-05 |
| SinvOR242 | 42.24 | 46.38 | 44.31 | -0.11 | 6.97E-01 | 4.68E-01 |
| SinvOR243 | 41.33 | 41.88 | 41.61 | -0.02 | 9.46E-01 | 8.82E-01 |
| SinvOR244 | 29.58 | 27.67 | 28.63 | 0.11 | 6.52E-01 | 3.91E-01 |
| SinvOR245 | 20.27 | 18.52 | 19.39 | 0.11 | 7.95E-01 | 6.07E-01 |
| SinvOR246 | 1.81 | 2.52 | 2.16 | -0.50 | 8.52E-01 | 7.00E-01 |
| SinvOR247 | 133.44 | 156.13 | 144.78 | -0.21 | 2.17E-01 | 3.34E-02 |
| SinvOR248 | 24.56 | 26.24 | 25.40 | -0.12 | 6.90E-01 | 4.56E-01 |
| SinvOR249 | 77.48 | 92.45 | 84.97 | -0.26 | 9.39E-02 | 4.91E-03 |
| SinvOR250 | 21.35 | 24.55 | 22.95 | -0.21 | 4.60E-01 | 1.77E-01 |
| SinvOR251 | 29.20 | 35.85 | 32.53 | -0.27 | 2.76E-01 | 6.12E-02 |
| SinvOR252 | 2.36 | 20.90 | 11.63 | -3.16 | 1.51E-02 | 1.86E-04 |
| SinvOR253 | 3.94 | 2.57 | 3.25 | 0.60 | 8.54E-01 | 7.07E-01 |
| SinvOR254 | 2.61 | 3.35 | 2.98 | -0.41 | 8.45E-01 | 6.81E-01 |
| SinvOR255 | 21.58 | 18.84 | 20.21 | 0.22 | 5.59E-01 | 2.79E-01 |
| SinvOR256 | 8.75 | 8.42 | 8.59 | 0.11 | 8.25E-01 | 6.49E-01 |
| SinvOR257 | 24.13 | 29.01 | 26.57 | -0.28 | 3.26E-01 | 8.82E-02 |
| SinvOR258 | 20.22 | 16.81 | 18.51 | 0.21 | 5.15E-01 | 2.33E-01 |
| SinvOR259 | 0.00 | 6.23 | 3.11 | -5.24 | 4.79E-02 | 1.77E-03 |
| SinvOR260 | 23.33 | 21.04 | 22.19 | 0.19 | 5.59E-01 | 2.77E-01 |
| SinvOR261 | 0.46 | 1.98 | 1.22 | -2.05 | 4.51E-01 | 1.68E-01 |
| SinvOR262 | 170.76 | 204.40 | 187.58 | -0.25 | 1.69E-01 | 1.87E-02 |
| SinvOR263 | 6.17 | 20.27 | 13.22 | -1.68 | 1.89E-02 | 4.08E-04 |
| SinvOR264 | 22.50 | 24.69 | 23.60 | -0.11 | 7.67E-01 | 5.69E-01 |
| SinvOR265 | 62.77 | 74.14 | 68.46 | -0.21 | 3.70E-01 | 1.18E-01 |
| SinvOR267 | 51.68 | 46.04 | 48.86 | 0.17 | 4.71E-01 | 1.87E-01 |
| SinvOR268 | 46.89 | 62.11 | 54.50 | -0.37 | 2.09E-01 | 3.09E-02 |
| SinvOR269 | 21.06 | 27.84 | 24.45 | -0.35 | 3.72E-01 | 1.21E-01 |
| SinvOR270 | 10.10 | 9.50 | 9.80 | 0.09 | 8.71E-01 | 7.45E-01 |
| SinvOR271 | 62.37 | 74.25 | 68.31 | -0.25 | 2.20E-01 | 3.45E-02 |
| SinvOR272 | 5.83 | 7.40 | 6.62 | -0.31 | 6.23E-01 | 3.51E-01 |
| SinvOR273 | 1.65 | 19.45 | 10.55 | -3.56 | 1.36E-01 | 1.13E-02 |

| | | | | | | |
|---|---|---|---|---|---|---|
| SinvOR274 | 25.15 | 18.71 | 21.93 | 0.44 | 5.34E-01 | 2.46E-01 |
| SinvOR275 | 9.84 | 2.52 | 6.18 | 1.91 | 3.09E-01 | 7.90E-02 |
| SinvOR276 | 8.22 | 6.99 | 7.61 | 0.26 | 7.09E-01 | 4.77E-01 |
| SinvOR277 | 9.81 | 10.02 | 9.91 | -0.03 | 9.62E-01 | 9.15E-01 |
| SinvOR278 | 8.33 | 10.83 | 9.58 | -0.34 | 6.56E-01 | 4.02E-01 |
| SinvOR279 | 32.72 | 36.81 | 34.76 | -0.18 | 4.87E-01 | 2.04E-01 |
| SinvOR281 | 4.53 | 5.44 | 4.98 | -0.30 | 8.51E-01 | 6.97E-01 |
| SinvOR282 | 18.02 | 19.60 | 18.81 | -0.14 | 7.79E-01 | 5.87E-01 |
| SinvOR283 | 19.17 | 17.03 | 18.10 | 0.13 | 7.14E-01 | 4.88E-01 |
| SinvOR284 | 8.82 | 5.00 | 6.91 | 0.82 | 4.76E-01 | 1.92E-01 |
| SinvOR285 | 2.81 | 6.06 | 4.43 | -1.13 | 5.50E-01 | 2.62E-01 |
| SinvOR286 | 0.70 | 1.55 | 1.13 | -1.20 | 7.24E-01 | 5.08E-01 |
| SinvOR287 | 25.65 | 21.80 | 23.72 | 0.22 | 5.82E-01 | 2.97E-01 |
| SinvOR289 | 0.47 | 1.96 | 1.22 | -2.06 | 4.41E-01 | 1.61E-01 |
| SinvOR290 | 4.53 | 7.03 | 5.78 | -0.67 | 6.51E-01 | 3.89E-01 |
| SinvOR291 | 2.69 | 3.93 | 3.31 | -0.53 | 7.91E-01 | 6.01E-01 |
| SinvOR292 | 17.36 | 14.52 | 15.94 | 0.25 | 5.73E-01 | 2.88E-01 |
| SinvOR293 | 7.20 | 8.05 | 7.62 | -0.11 | 8.61E-01 | 7.18E-01 |
| SinvOR294 | 1.52 | 0.00 | 0.76 | 2.90 | 3.97E-01 | 1.38E-01 |
| SinvOR295 | 14.00 | 16.70 | 15.35 | -0.27 | 4.92E-01 | 2.12E-01 |
| SinvOR296 | 1.25 | 1.92 | 1.59 | -0.60 | 8.58E-01 | 7.12E-01 |
| SinvOR297 | 6.07 | 12.97 | 9.52 | -1.11 | 2.96E-01 | 7.19E-02 |
| SinvOR298 | 2.58 | 2.93 | 2.76 | -0.15 | 8.80E-01 | 7.56E-01 |
| SinvOR299 | 32.91 | 26.25 | 29.58 | 0.36 | 3.34E-01 | 9.67E-02 |
| SinvOR300 | 36.52 | 35.52 | 36.02 | 0.08 | 7.70E-01 | 5.75E-01 |
| SinvOR301 | 15.34 | 12.47 | 13.90 | 0.32 | 5.28E-01 | 2.42E-01 |
| SinvOR302 | 24.44 | 23.32 | 23.88 | 0.09 | 7.58E-01 | 5.48E-01 |
| SinvOR304 | 4.94 | 5.27 | 5.11 | -0.16 | 8.38E-01 | 6.68E-01 |
| SinvOR305 | 17.11 | 11.18 | 14.14 | 0.56 | 2.76E-01 | 6.03E-02 |
| SinvOR306 | 35.10 | 30.34 | 32.72 | 0.20 | 5.46E-01 | 2.55E-01 |
| SinvOR307 | 6.10 | 5.67 | 5.88 | 0.03 | 9.59E-01 | 9.06E-01 |
| SinvOR308 | 23.44 | 26.19 | 24.81 | -0.13 | 6.37E-01 | 3.64E-01 |
| SinvOR309 | 25.88 | 28.07 | 26.98 | -0.18 | 6.23E-01 | 3.49E-01 |
| SinvOR312 | 12.58 | 3.16 | 7.87 | 1.94 | 2.73E-01 | 5.88E-02 |
| SinvOR313 | 23.45 | 18.18 | 20.82 | 0.36 | 2.50E-01 | 4.78E-02 |
| SinvOR314 | 14.33 | 18.88 | 16.61 | -0.36 | 5.50E-01 | 2.61E-01 |
| SinvOR315 | 8.92 | 8.67 | 8.80 | -0.02 | 9.78E-01 | 9.57E-01 |
| SinvOR316 | 11.62 | 15.40 | 13.51 | -0.40 | 8.85E-01 | 7.68E-01 |
| SinvOR317 | 16.23 | 14.79 | 15.51 | 0.18 | 6.22E-01 | 3.46E-01 |
| SinvOR318 | 43.74 | 42.65 | 43.19 | 0.03 | 9.08E-01 | 8.18E-01 |
| SinvOR319 | 6.30 | 3.29 | 4.79 | 0.87 | 3.10E-01 | 8.02E-02 |
| SinvOR320 | 7.21 | 6.69 | 6.95 | 0.15 | 8.33E-01 | 6.59E-01 |

| | | | | | | |
|---|---|---|---|---|---|---|
| SinvOR321 | 3.44 | 2.09 | 2.76 | 0.79 | 4.90E-01 | 2.08E-01 |
| SinvOR322 | 5.77 | 11.80 | 8.79 | -1.03 | 5.48E-02 | 2.19E-03 |
| SinvOR323 | 82.44 | 93.88 | 88.16 | -0.17 | 3.63E-01 | 1.13E-01 |
| SinvOR325 | 17.31 | 5.51 | 11.41 | 1.72 | 8.71E-03 | 8.04E-05 |
| SinvOR326 | 66.96 | 38.75 | 52.85 | 0.76 | 3.12E-02 | 8.64E-04 |
| SinvOR327 | 6.29 | 9.73 | 8.01 | -0.60 | 2.97E-01 | 7.31E-02 |
| SinvOR328 | 22.23 | 21.52 | 21.88 | 0.04 | 9.19E-01 | 8.34E-01 |
| SinvOR329 | 1.75 | 3.90 | 2.82 | -1.17 | 2.69E-01 | 5.45E-02 |
| SinvOR330 | 21.58 | 17.76 | 19.67 | 0.33 | 3.96E-01 | 1.36E-01 |
| SinvOR331 | 11.28 | 6.70 | 8.99 | 0.74 | 3.68E-01 | 1.16E-01 |
| SinvOR332 | 2.68 | 1.33 | 2.00 | 1.03 | 4.04E-01 | 1.42E-01 |
| SinvOR333 | 6.76 | 6.72 | 6.74 | -0.02 | 9.87E-01 | 9.75E-01 |
| SinvOR334 | 8.41 | 5.49 | 6.95 | 0.54 | 4.67E-01 | 1.84E-01 |
| SinvOR335 | 2.99 | 2.14 | 2.57 | 0.42 | 7.21E-01 | 5.01E-01 |
| SinvOR336 | 3.14 | 2.54 | 2.84 | 0.23 | 8.15E-01 | 6.40E-01 |
| SinvOR337 | 34.56 | 41.87 | 38.21 | -0.26 | 3.35E-01 | 9.79E-02 |
| SinvOR338 | 3.57 | 6.64 | 5.10 | -0.92 | 1.78E-01 | 2.24E-02 |
| SinvOR339 | 1.10 | 1.77 | 1.43 | -0.76 | 5.98E-01 | 3.22E-01 |
| SinvOR340 | 101.59 | 87.41 | 94.50 | 0.23 | 3.23E-01 | 8.54E-02 |
| SinvOR342 | 26.74 | 23.76 | 25.25 | 0.14 | 6.58E-01 | 4.05E-01 |
| SinvOR343 | 56.40 | 53.51 | 54.96 | 0.09 | 7.18E-01 | 4.95E-01 |
| SinvOR344 | 33.66 | 32.98 | 33.32 | 0.06 | 8.62E-01 | 7.24E-01 |
| SinvOR345 | 54.64 | 69.24 | 61.94 | -0.31 | 2.61E-01 | 5.22E-02 |
| SinvOR346 | 7.14 | 6.15 | 6.64 | 0.22 | 7.81E-01 | 5.91E-01 |
| SinvOR347 | 12.36 | 15.85 | 14.10 | -0.30 | 5.58E-01 | 2.74E-01 |
| SinvOR348 | 26.14 | 18.19 | 22.16 | 0.51 | 1.68E-01 | 1.81E-02 |
| SinvOR349 | 32.61 | 28.24 | 30.43 | 0.20 | 4.30E-01 | 1.55E-01 |
| SinvOR350 | 33.81 | 34.01 | 33.91 | -0.03 | 9.15E-01 | 8.28E-01 |
| SinvOR351 | 25.10 | 33.67 | 29.38 | -0.40 | 1.50E-01 | 1.48E-02 |
| SinvOR352 | 41.70 | 45.97 | 43.84 | -0.13 | 6.26E-01 | 3.56E-01 |
| SinvOR353 | 37.27 | 34.16 | 35.72 | 0.14 | 5.56E-01 | 2.72E-01 |
| SinvOR354 | 13.40 | 23.49 | 18.45 | -0.76 | 9.81E-02 | 5.43E-03 |
| SinvOR355 | 10.54 | 12.78 | 11.66 | -0.32 | 6.63E-01 | 4.14E-01 |
| SinvOR356 | 51.50 | 50.48 | 50.99 | 0.05 | 8.46E-01 | 6.84E-01 |

**Table S4** Primers used for RT-PCR

| Primer | Sequences (5'to3') |
|---|---|
| SinvOrco-F | TCCAATCACGAACGAGACGG |
| SinvOrco-R | CAGTGGCAAGAATAAGCCGC |
| SinvOR89-F | ATCTGGCCGCTTTATCGAGG |
| SinvOR89-R | CACGCAGGAGGCAATTAAGC |
| SinvOR102-F | GTTTCCAGTGCCTAGCGTGA |
| SinvOR102-R | AACATCACCGAAGCTGCTGA |
| SinvOR352-F | TGTCCTGCTGCTACAAGTGG |
| SinvOR352-R | CTTCCGGCGGTTGTTTGATG |
| SinvOR327-F | CCCAGGAACGAACACGAAAC |
| SinvOR327-R | GGCCGATTACTGTCGAGCTTA |
| SinvOR135-F | GCCTCATCTTCATGCTGGGT |
| SinvOR135-R | TTGAGGTGATGTTGCGCTCT |
| SinvRpl18-F | AAGCATGATCGGAAAGTGCG |
| SinvRpl18-R | TTTCAGGATACGTGCCCGAG |

## 1.2 Supplementary Figures

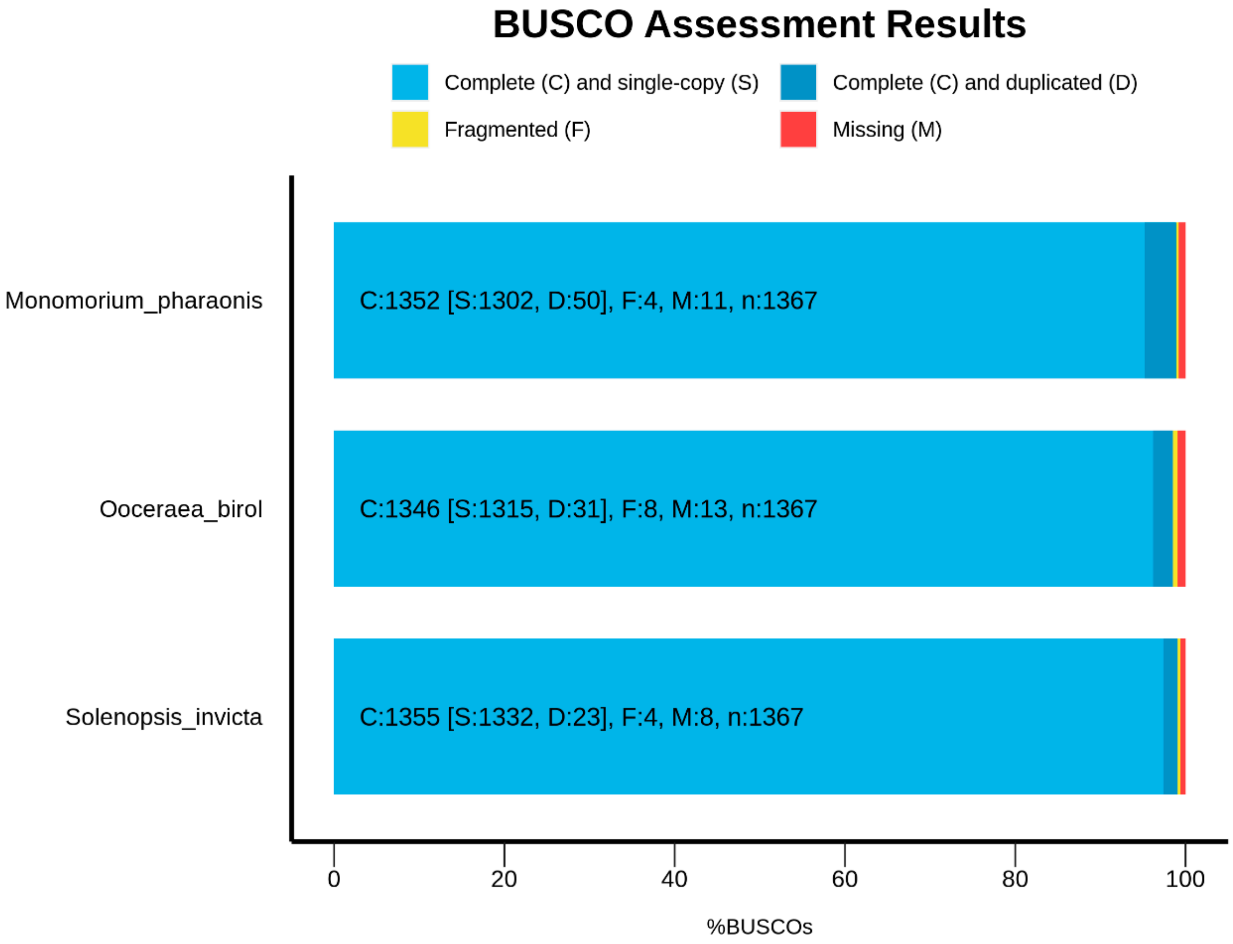


**Figure S1**. BUSCO assessment of the three Formicidae species. Data were download from NCBI: *M. pharaonis* (GCF_013373865.1), *O. biroi* (GCF_003672135.1), *S. invicta* (GCF_016802725.1).

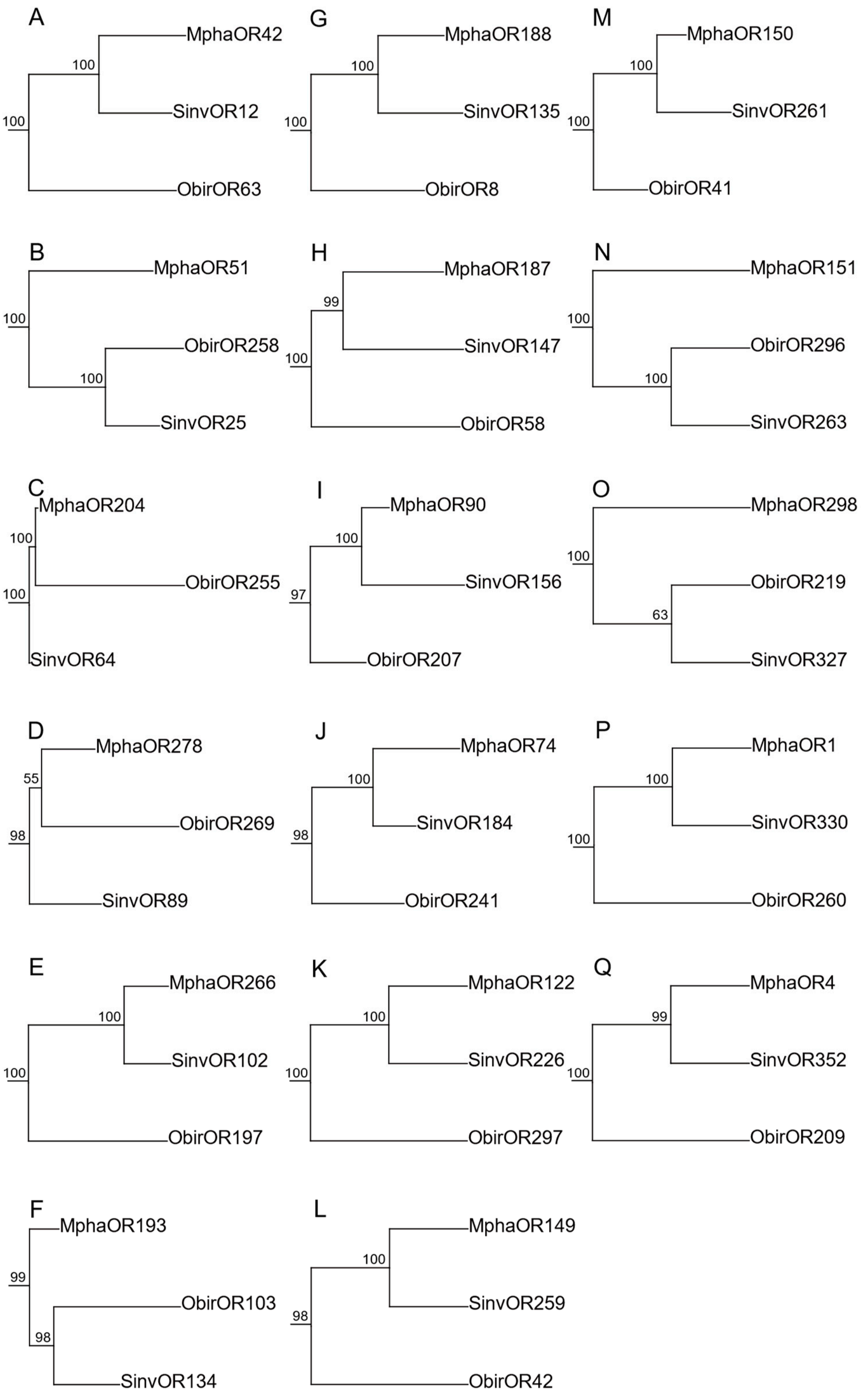


**Figure S2**. Single-copy orthologous branch of the three Formicidae species.

## Reference

Wang, Y., Tang, H., DeBarry, J.D., Tan, X., Li, J., Wang, X., et al. (2012). MCScanX: a toolkit for detection and evolutionary analysis of gene synteny and collinearity. *Nucleic Acids Research* 40(7)**,** e49-e49. doi: 10.1093/nar/gkr1293.
